# Performance of Predefined Viability Criteria During Prolonged End‐Ischemic Normothermic Perfusion of Brain‐Dead Donor Liver Grafts

**DOI:** 10.1111/ctr.70539

**Published:** 2026-04-25

**Authors:** Heithem Jeddou, Stylianos Tzedakis, Michel Samson, Karim Boudjema

**Affiliations:** ^1^ Department of Hepatobiliary and Digestive Surgery Rennes University Hospital University of Rennes Rennes France; ^2^ INSERM UMR 1085, IRSET (Institut de Recherche en Santé, Environnement et Travail), EHESP University of Rennes Rennes France; ^3^ Department of Digestive, Hepatobiliary and Endocrine Surgery Cochin Hospital, AP‐HP Université Paris Cité Paris France

**Keywords:** biliary complications, brain‐death donors, early allograft dysfunction, liver transplantation, normothermic machine perfusion, viability criteria

## Abstract

**Background:**

Normothermic machine perfusion (NMP) maintains physiological metabolism ex situ and enables functional assessment of liver grafts. However, the clinical performance of predefined viability criteria in liver transplantation from brain‐death donors (DBD) remains uncertain.

**Methods:**

This single‐center study, an ancillary of the OVERNIGHT NMP program (Rennes University Hospital, 2021–2023), evaluated predefined viability thresholds applied during prolonged end‐ischemic NMP. All adult DBD grafts retrieved between 16:00 and 00:00 underwent NMP until scheduled transplantation the following morning. Grafts meeting predefined viability criteria were transplanted. Outcomes were compared with 1:3 propensity‐matched controls preserved by static cold storage (SCS) in exploratory, hypothesis‐generating analyses. The primary endpoint was early allograft dysfunction (EAD); a modified definition (mEAD, excluding transaminase criteria) was used as a sensitivity analysis. Secondary endpoints included primary non‐function (PNF), biliary and vascular complications, and death‐censored graft survival.

**Results:**

Among 382 liver transplants, 18 NMP and 54 matched SCS recipients were analyzed. All 18 NMP grafts fulfilling predefined thresholds were transplanted, while two non‐viable grafts were discarded. No PNF occurred. EAD and mEAD rates were comparable between NMP and SCS (44.4% vs. 26.0%, *p* = 0.204; 17% vs. 24%, *p* = 0.550). Biliary complications, requiring endoscopic or surgical intervention, were similar (43.8% vs. 36.6%, *p* = 0.684). Death‐censored graft survival at 24 months did not differ (88.5% vs. 96.0%, *p* = 0.584).

**Conclusions:**

Predefined viability criteria applied during NMP allowed safe identification of transplantable DBD grafts. However, functional assessment based solely on hepatocellular markers was insufficient to anticipate biliary complications, underscoring the need for integrative viability algorithms incorporating cholangiocellular or molecular indicators.

**Abbreviations**
aHR: adjusted Hazard ratios
ALT: alanine aminotransferase
AST: aspartate aminotransferase
BMI: body mass index
CD: Clavien‐Dindo
CI: confidence intervals
CIT: cold ischemia time
DBD: brain‐death donors
DCD: donation‐after‐circulatory‐death
EAD: early allograft dysfunction
ECD: extended‐criteria donor
HCC: hepatocellular carcinoma
HR: hazards Ratio
ICU: intensive care unit
LT: liver transplantation
MASLD: metabolic dysfunction‐associated steatotic liver disease
mEAD: modified Early allograft dysfunction
MELD: model for end‐stage liver disease
NMP: normothermic machine perfusion
PNF: primary non‐function
POD: post operative day
SCS: static cold storage
SMD: standardized mean differences
TIPS: transjugular intrahepatic portosystemic shunt

## 1 Introduction

Normothermic machine perfusion (NMP) reproduces near‐physiological conditions *ex vivo*, maintaining aerobic metabolism and enabling continuous biochemical and hemodynamic monitoring [1]. It is increasingly applied to preserve and assess extended‐criteria donor (ECD) liver grafts before transplantation, with the goal of safely expanding the donor pool [2, 3, 4, 5]. Functional assessment during NMP relies on biochemical markers of hepatocellular and cholangiocellular activity. Hepatocellular function is mainly evaluated through lactate clearance, bile production, and transaminase release, whereas biliary function is assessed by bile composition (pH, bicarbonate, and glucose) [6, 7, 8, 9, 10, 11]. Combining these parameters may improve graft selection and reduce posttransplant biliary complications [12, 13, 14, 15].

Previous studies have shown that livers fulfilling predefined viability thresholds, particularly rapid lactate clearance, sustained bile production, and stable perfusate pH, can be transplanted safely, even in high‐risk or previously discarded grafts [16, 17, 18, 19, 20]. Real‐world evidence confirms that NMP increases organ utilization without compromising early graft function [12, 21]. However, current thresholds remain largely empirical and may not fully reflect the liver's adaptive capacity, leading to both unnecessary discards and posttransplant graft dysfunction [8].

The OVERNIGHT NMP program was developed to maintain normothermic preservation until planned transplantation the following morning after late‐hour donor retrievals. This clinical setting provided an opportunity to assess the feasibility and clinical safety of applying predefined viability criteria during prolonged end‐ischemic NMP in DBD liver transplantation. The present study therefore evaluated the clinical performance of a predefined viability algorithm by comparing early posttransplant outcomes of grafts meeting these criteria with matched static cold storage (SCS) controls.

## 2 Materials and Methods

### 2.1 Study Design and Ethical Approval

This study is an ancillary analysis of the OVERNIGHT NMP program conducted at Rennes University Hospital between March 2021 and July 2023. The original protocol aimed to assess the logistical feasibility and clinical safety of prolonged end‐ischemic NMP allowing transplantation to be postponed to the following morning after late donor retrievals. Although primarily designed for logistical optimization rather than high‐risk graft rescue, the program provided a controlled setting to evaluate graft function before implantation. The present analysis specifically focused on the clinical performance of predefined viability criteria applied during these perfusions, by comparing posttransplant outcomes of viable NMP grafts with a propensity‐matched control group preserved by SCS during the same period.

The study protocol was approved by the institutional ethics committee (University Hospital Rennes, approval No. 25.139) and complied with French regulations for research involving human biological material (MR‐004). Written informed consent for the use of donor tissue and recipient clinical data was obtained in accordance with national guidelines.

### 2.2 Donor Grafts and Perfusion Protocol

After retrieval, all livers were transported in SCS to our center and prepared for NMP using the OrganOx Metra system, which maintains the liver at 37°C under continuous dual perfusion (hepatic artery and portal vein) with automated control of flow, pressure, temperature, and oxygenation. The perfusate consisted of Gelofusine, three units of donor‐matched packed red blood cells, insulin, prostacyclin, heparin, calcium gluconate, antibiotics, and parenteral nutrition (Nutriflex). Perfusion duration ranged from 4 to 24 h according to surgical scheduling.

Perfusate blood gas analyses (including lactate and glucose) were performed hourly in the central biochemistry laboratory. In parallel, perfusate pH, oxygenation, hemodynamic parameters (arterial and portal flows and pressures), and bile production were continuously recorded by the device. Detailed procurement procedures, perfusion settings, surgical technique and postoperative management, are provided in the Supporting Information.

### 2.3 Preservation Time Definitions

In the NMP group, cold ischemia time (CIT) was defined as the interval between organ retrieval and initiation of NMP. No secondary cold ischemia occurred after termination of NMP, as graft implantation followed immediately. Total preservation time for NMP grafts was therefore defined as the sum of CIT before NMP and NMP duration.

In the SCS group, total preservation time corresponded to total CIT.

### 2.4 Viability Assessment

Graft viability was assessed at 240 min according to predefined criteria (Table 1) [11]. These included: (1) perfusate lactate ≤ 2.5 mmol/L; (2) sustained bile production; (3) perfusate pH ≥ 7.30; (4) evidence of glucose consumption; and (5) stable hemodynamic flows (arterial ≥ 150 mL/min, portal ≥ 500 mL/min). A graft was considered suitable for transplantation if lactate clearance criterion was met together with at least two of the remaining parameters. Sustained bile production was defined as the presence of continuous bile secretion observed during viability assessment at 240 min of NMP, with no predefined minimum bile volume in the study protocol. Cholangiocellular viability parameters (bile pH, bicarbonate, and glucose) were not incorporated into the predefined assessment protocol. Although biliary complications remain clinically relevant in brain‐death donor (DBD), such markers are currently better validated in donation‐after‐circulatory‐death (DCD) grafts, and their role in routine DBD graft assessment remains less clearly defined [22, 23].

**TABLE 1:** Propensity score matched cohort covariate balancing between normothermic machine perfusion and static cold storage for patients undergoing orthotopic liver transplantation.

Characteristics	Overall *N* = 72^a^	SCS *N* = 54^a^	NMP *N* = 18^a^	Difference (95% CI)^b^, ^c^	*p*‐value^a^
**Recipient patient characteristics**					
**Age (years)** ^d^, ^f^	61 (54, 65)	61 (54, 65)	61 (52, 67)	0.02 (−0.52, 0.55)	0.958^e^
**Sex** ^f^				0.07 (−0.47, 0.60)	>0.999
Female	18 (25)	14 (26)	4 (22)		
Male	54 (75)	40 (74)	14 (78)		
**Smoking**	42 (58)	30 (56)	12 (67)	−0.23 (−0.76, 0.31)	0.408
**Diabetes type 2**	24 (33)	16 (29)	8 (44)	−0.32 (−0.86, 0.21)	0.229
**Arterial hypertension**	24 (34)	16 (30)	8 (44)	−0.30 (−0.83, 0.24)	0.269
**Indication for LT** ^f^				0.07 (−0.47, 0.60)	>0.999
Cirrhosis	67 (93)	50 (93)	17 (94)		
HCC	5 (6.9)	4 (7.4)	1 (5.6)		
**Cirrhosis, aetiology** ^f^				0.21 (−0.32, 0.74)	0.706
Alcohol	54 (75)	41 (76)	13 (72)		
MASLD	5 (6.9)	3 (5.6)	2 (11)		
Other	13 (18)	10 (19)	3 (17)		
**MELD score** ^f^	25 (17, 30)	26 (18, 32)	20 (15, 29)	0.51 (−0.03, 1.1)	0.091^e^
**Ascites before LT** ^f^	52 (74)	39 (75)	13 (72)	0.06 (−0.47, 0.60)	>0.999
**Portal hypertension** ^f^				0.47 (−0.06, 1.0)	0.168
Absent	13 (18)	9 (17)	4 (22)		
Moderate	47 (65)	38 (70)	9 (50)		
Severe	12 (17)	7 (13)	5 (28)		
**TIPS** ^f^	5 (6.9)	3 (5.6)	2 (11)	−0.20 (−0.74, 0.33)	0.593
**Bridge treatment to LT**	10 (14)	9 (17)	1(5.6)	0.36 (−0.18, 0.90)	0.434
**ABO Blood Group**				0.62(0.07, 1.2)	0.157
A	31 (43)	26 (48)	5(28)		
AB	2 (2.8)	1 (1.9)	1(5.6)		
B	12 (17)	10 (19)	2(11)		
O	27 (38)	17 (31)	10 (56)		
**Donor characteristics**					
**Age, years** ^b^, ^f^	69 (58, 80)	69 (58, 81)	69 (56, 74)	−0.01 (−0.50, 0.56)	0.566^e^
**Age >65 years**	48 (65)	36 (64)	12 (67)	−0.05 (−0.55, 0.52)	0.682
**Sex** ^f^				0.12 (−0.42, 0.65)	0.673
Female	27 (38)	21 (39)	6 (33)		
Male	45 (62)	33 (61)	12 (67)		
**BMI** ^f^	26 (23, 28)	26 (23, 28)	26 (23, 27)	−0.01 (−0.54, 0.52)	0.979^e^
**BMI >30** ^f^	10 (14)	8 (15)	2 (11)	0.11 (−0.42, 0.64)	>0.999
**Liver macrovesicular steatosis** ^d^	5 (0, 10)	3 (0, 13)	5 (5, 10)	−0.10 (−0.65, 0.46)	0.280
**Liver macrovesicular steatosis > 30%** ^f^	8 (11)	7 (13)	1 (5.6)	0.24 (−0.29, 0.78)	0.670
**AST >150**	6 (8.3)	5 (9.3)	1 (5.6)	0.14 (−0.39, 0.68)	>0.999
**ALT >170**	7 (9.7)	5 (9.3)	2(11)	−0.06 (−0.59, 0.47)	>0.999
**Sodium (serum) >155 mmol/L** ^f^	9 (13)	5 (9.3)	4 (22%)	−0.36 (−0.90,0.17)	0.214
**Occurrence of cardiac arrest before liver procurement** ^f^	8 (11)	5 (9.3%)	3 (17)	−0.22 (−0.76, 0.31)	0.404
**ICU stay prior to liver procurement >7 days** ^f^	0 (0)	0 (0)	0 (0)	0.0 (−0.53, 0.53)	—
**Extended criteria donor** ^f^	63 (88)	47 (87)	16 (89)	−0.06 (−0.59, 0.48)	>0.999

*Note:* Balance of matching variables after inverse probability of treatment weighting was assessed using the standard mean difference (SMD). SMD value ≤ 0.1 indicates very small differences; value between 0.101 and 0.300 indicates small differences; value between 0.301 and 0.500 indicates moderate differences; value above 0.500 indicates considerable differences.

Abbreviations: ALT, alanine aminotransferase; AST, aspartate aminotransferase; BMI, body mass index; HCC, hepatocellular carcinoma; ICU, intensive care unit; LT, liver transplantation; MASLD, metabolic dysfunction‐associated steatotic liver disease; MELD, model for end‐stage liver disease; NMP, normothermic machine perfusion; TIPS, transjugular intrahepatic portosystemic shunt.

^a^ Values are expressed with percentages in parentheses unless indicated otherwise; *p*‐values are from a McNemar's test for paired data unless indicated otherwise.

^b^ Standardized Mean Difference.

^c^ CI = Confidence Interval.

^d^ Values are median (IQR).

^e^ Wilcoxon Signed‐Rank test for paired data.

^f^ Indicating variables used for the propensity score patient matching.

### 2.5 Inclusion Criteria

#### 2.5.1 NMP Group

All adult liver‐only transplantations performed at Rennes University Hospital between March 2021 and July 2023 were screened for eligibility. Patients were included if they received a DBD graft in which hepatectomy was completed between 16:00 and 00:00, qualifying the organ for OVERNIGHT NMP. According to the protocol, the recipient operation was systematically scheduled to start the following morning at or after 8:00 a.m., depending on operating room availability.

#### 2.5.2 Control Group

The control group was selected using 1:3 propensity score matching among adult DBD liver transplantations (LT) performed at the same center and during the same period using SCS. Matching variables included donor age, sex, body mass index (BMI), extended‐criteria donor (ECD) status, and CIT, as well as recipient age, sex, MELD score, indication for transplantation, etiology of liver disease, presence of ascites, severity of portal hypertension, and preoperative transjugular intrahepatic portosystemic shunt (TIPS) use.

### 2.6 Exclusion Criteria

Retransplantation, acute liver failure, combined or split LT, and use of hypothermic machine perfusion were excluded from the analysis. DCD grafts were excluded, as they are managed by normothermic regional perfusion and are not eligible for ex situ NMP in France.

### 2.7 Study Endpoints and Outcome Measures

The primary endpoint of this study was the incidence of EAD as defined according to Olthoff criteria [24] (bilirubin ≥10 mg/dL on postoperative day 7, INR ≥1.6 on day 7, or AST or ALT >2000 IU/L within the first 7 days). Because elevated transaminases may result from localized graft compression against the NMP tray rather than reflecting genuine hepatocellular injury, as previously described [25, 26], a modified EAD definition (mEAD), excluding transaminases criteria and considering only bilirubin ≥ 10 mg/dL and/or INR ≥ 1.6 on postoperative day 7, was pre‐specified and used as a sensitivity analysis to account for early transaminase elevations potentially related to the NMP device rather than intrinsic graft dysfunction.

Secondary endpoints included the incidence of PNF, defined as liver failure requiring re‐transplantation or leading to death within 7 days after transplantation [27], and the incidence of biliary complications, including bile leak and both anastomotic and non‐anastomotic strictures. Vascular complications were also recorded. Graft outcomes included overall and death‐censored graft survival, the latter defined as graft survival censored for patient death and analyzed with death as a competing risk. Postoperative morbidity was assessed according to the Clavien‐Dindo (CD) classification within 90 days after transplantation, with severe morbidity defined as CD ≥ III [28]. Finally, the duration of intensive care unit (ICU) and total hospital stay were analyzed as additional secondary outcomes.

### 2.8 Statistical Analysis

Continuous variables were expressed as medians with 25–75 interquartile range (IQR) and compared using the Wilcoxon Signed‐Rank test for matched samples. Categorical variables were summarized as frequency/ percentage and compared using the McNemar's test. Patient matching was based on a 1:3 nearest neighbor matching (caliper restriction adjusted at 0.2) without replacement according to the propensity score and the balance of matching variables was assessed using standardized mean differences (SMD). SMD values ≤ 0.1 indicated very small differences; values between 0.101 and 0.300 indicated small differences; values between 0.301 and 0.500 indicated moderate differences and values above 0.500 indicated considerable differences. Death‐censored graft survival was calculated from the time of LT to the time of graft loss or last follow‐up and compared using Kaplan–Meier curves and cause‐specific multivariable Cox proportional hazards model for graft failure (death‐censored). Competing risk analysis was also used to estimate the probability of graft loss in the presence of the competing risks of patient death to avoid overestimating graft survival. The association of variables with death‐censored graft loss was calculated using multivariable Cox‐proportional hazards models, Fine and Gray models and associations were expressed using adjusted Hazard ratios (aHR) and subhazard ratios with their 95% confidence intervals (CI), respectively. Covariate multi‐collinearity was assessed with a variance inflation factor, proportional hazards assumptions were tested using Schoenfeld residuals. All models were adjusted for known risk factors of graft failure and variables included in the univariable Cox or Fine and Gray analyses and presenting a *p*‐value < 0.2. Missing data were managed using the multiple imputation chained methodology, all models were performed in each imputed data set (*n* = 20), and the estimates and standard errors were pooled into a final point estimate with robust standard errors according to Rubin's rule.29 All statistical tests were based on two‐tailed *p*‐values, with *p* < 0.05 considered to indicate statistical significance. All analyses were performed using RStudio statistical software (Version 2024.04.2+764 2024 RStudio, Inc). Given the limited sample size and low number of graft‐loss events, survival analyses were primarily descriptive, with emphasis on Kaplan–Meier curves and cumulative incidence functions, and multivariable analyses were restricted to parsimonious models consistent with the observed number of events.

Missing data were limited across variables, with no variable having more than 5% missing values. Multiple imputation by chained equations was therefore used to limit potential bias, and sensitivity analyses comparing imputed and complete‐case analyses yielded consistent results.

## 3 Results

Between March 1, 2021, and July 31, 2023, a total of 382 LT were performed at our center. Among these, 18 grafts were preserved using end‐ischemic NMP and 364 using SCS. After applying predefined exclusion criteria, 274 SCS recipients constituted the pool of potential controls. Following 1:3 nearest‐neighbor propensity score matching, 54 SCS recipients were matched to the 18 NMP recipients (Figure S1).

Baseline donor and recipient characteristics are summarized in Table 1. The median donor age was 69 years (IQR 58–80), and 62% were male. The median recipient age was 61 years (54–65), and 75% were male. The median MELD score was 25 (17–30). Cirrhosis accounted for 93% of indications, predominantly alcohol‐related (75%). After matching, donor and recipient characteristics were well balanced between the NMP and SCS groups, including ECD status. The median CIT was shorter in the NMP group than in SCS controls (320 [243–380] min vs. 369 [323–431] min, *p* = 0.020). Preservation times are summarized as medians with interquartile ranges in Table 2. Detailed graft‐level preservation time components for transplanted NMP grafts are provided in Table S1. Intra‐operative transfusion requirements and other peri‐operative parameters were comparable between groups (Table 2).

**TABLE 2:** Propensity score matched cohort intraoperative and postoperative outcomes between normothermic oxygenated machine perfusion and static cold storage for patients undergoing orthotopic liver transplantation.

Characteristic	Overall (*N* = 72)^a^	SCS (*N* = 54)^a^	NMP (*N* = 18)^a^	*p*‐value^a^
**Intraoperative parameters**				
**Cold ischemia time, minutes** ^d^	355 (312, 425)	369 (323, 431)	320 (243, 380)	**0.020** ^e^
**Liver perfusion time, minutes** ^d^	0 (0, 719)	0 (0, 0)	719 (614, 800)	—
**Red blood cell transfusion, units** ^d^	3 (0, 5)	3 (0, 5)	3 (0, 4)	0.560^e^
**Postoperative and long‐term outcomes**				
**Early allograft dysfunction** ^f^	22 (30.6)	14 (26.0)	8 (44.4)	0.204
**Modified early allograft dysfunction (INR, bilirubin POD 7)** ^f^	16 (22)	13 (24)	3 (17)	0.550
**Peak AST (IU/L)**	467 (234, 785)	467 (234, 606)	470 (272, 1354)	**0.047**
**Peak ALT (IU/L)**	558 (367, 1004)	558 (400, 874)	404 (289, 2046)	0.149
**Primary non‐function** ^f^	0 (0.0)	0 (0.0)	0 (0.0)	—
**Retransplantation**	4 (6.2)	3 (6.3)	1 (5.6)	0.910
**Biliary complication**	27 (38.2)	20 (36.6)	7 (43.8)	0.684
**Arterial complication**	8 (11.1)	6 (11.1)	2 (11.1)	0.996
**Venous complication**	1 (1.4)	0 (0.0)	1 (5.6)	0.087
**Postoperative severe complications (Clavien‐Dindo ≥3)**	16 (21.8)	16 (29.1)	0 (0.0)	**0.027**
**Clavien‐Dindo 5 (Death)**	2 (2.3)	1 (1.2)	1 (5.6)	0.087
**ICU postoperative stay, days** ^d^	4 (2, 5)	4 (2, 6)	4 (2, 5)	0.605
**Overall postoperative stay, days** ^d^	16 (12, 33)	16 (12, 33)	14 (13, 28)	0.838^e^

*Note:* Balance of matching variables after inverse probability of treatment weighting was assessed using the standard mean difference (SMD). SMD value ≤ 0.1 indicates very small differences; value between 0.101 and 0.300 indicates small differences; value between 0.301 and 0.500 indicates moderate differences; value above 0.500 indicates considerable differences.

Abbreviations: ALT, alanine aminotransferase; AST, aspartate aminotransferase; ICU, intensive care unit; NMP, normothermic machine perfusion; POD, post operative day; SCS, static cold storage.

^a^ Values are expressed with percentages in parentheses unless indicated otherwise; *p*‐values are from a McNemar's test for paired data unless indicated otherwise.

^b^ Standardized Mean Difference.

^c^ CI = Confidence Interval.

^d^ Values are median (IQR).

^e^ Wilcoxon Signed‐Rank test for paired data.

^f^ Early allograft dysfunction according to the Olthof criteria, Modified EAD defined as meeting INR and bilirubin criteria only, and Primary allograft non function defined as liver failure requiring retransplantation or leading to death within 7 days after transplantation.

### 3.1 Perfusion Characteristics and Viability Assessment

NMP was maintained for a median of 719 min (IQR 614–800) (Table 2). All grafts underwent standardized evaluation at 4 h according to predefined viability criteria (Table S2). Among the 20 perfused livers, 18 (90%) fulfilled predefined thresholds and were transplanted, whereas 2 (10%) were discarded because of persistently elevated lactate.

**FIGURE 1:**
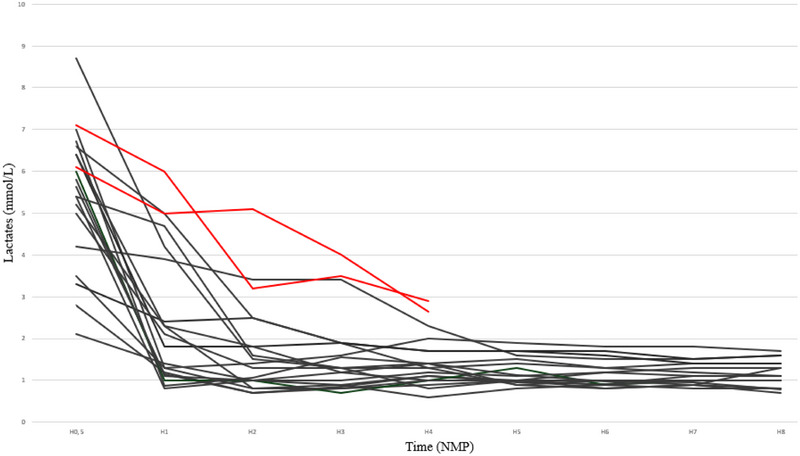
Lactate kinetics during NMP. Individual lactate trajectories for all perfused grafts are shown. Black lines represent grafts meeting predefined viability criteria and subsequently transplanted, demonstrating rapid lactate clearance within the first 2 h of NMP. Red lines correspond to grafts that failed to meet viability criteria and were discarded. H, hour; NMP, Normothermic Machine Perfusion.

Lactate decreased from an initial 6–7 mmol/L to ≤ 2.5 mmol/L within 2–3 h (Figure 1) and the median lactate level at 4 h was 1.3 mmol/L (0.6–2.3) (Table S3). The two discarded grafts had lactate levels of 2.64 and 2.89 mmol/L at 4 h of NMP. Perfusate pH remained stable across all viable grafts (7.33, [7.22–7.46]); the two non‐viable grafts had lower values (7.12 and 7.20).

Bile was produced in 13 of 18 transplanted grafts (72%) at 4 h. The absence of bile output was not associated with any case of PNF and did not coincide with a higher frequency of EAD. All viable grafts demonstrated active glucose consumption, with a median decrease of 3.8 mmol/L between perfusion start and 4 h, whereas the two non‐viable grafts showed stable or rising glucose levels. Arterial and portal flows remained stable and within physiological ranges in all cases (median 550 mL/min and 1100 mL/min, respectively).

Macroscopic inspection confirmed homogeneous perfusion and soft parenchymal consistency in all viable grafts. In contrast, the two non‐transplanted livers displayed patchy discoloration during perfusion (Figure S2) and histologically, they presented macrovesicular steatosis (50%–60%), F2 fibrosis, sinusoidal congestion, and focal necrosis. Minor technical incidents occurred in 4 perfusions (22%), caused by localized posterior compression on the perfusion tray; all resolved without sequelae; no device malfunction or bleeding was occurred (Figure S3).

### 3.2 Study Endpoints and Outcomes

#### 3.2.1 Postoperative Outcomes

**FIGURE 2:**
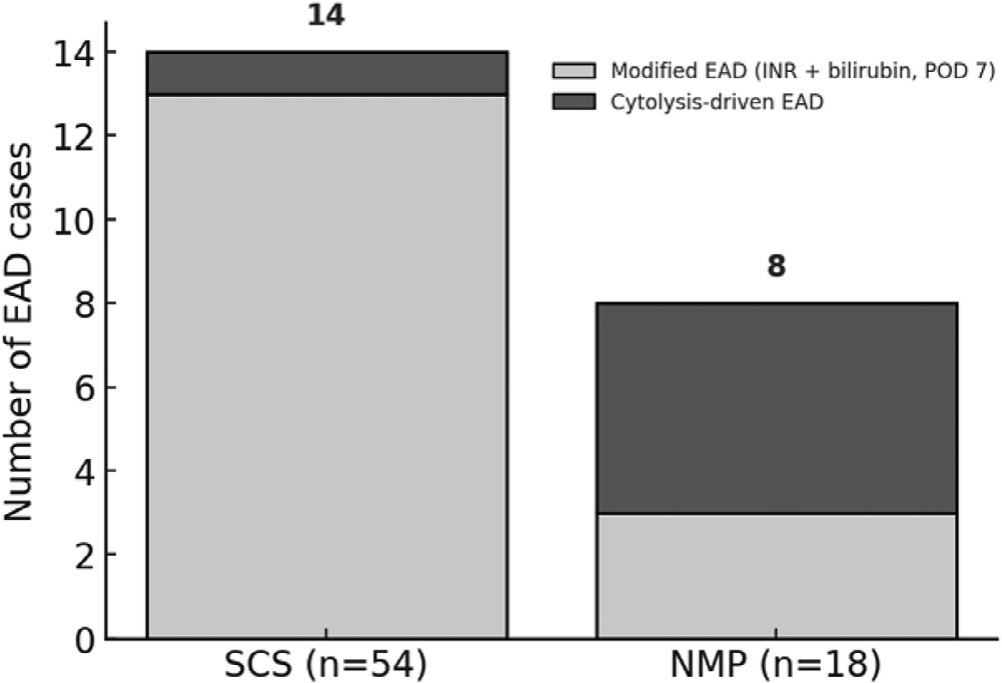
Distribution of EAD phenotypes by preservation method. SCS, static cold storage; NMP, normothermic machine perfusion; EAD, early allograft dysfunction; POD, postoperative day.

**FIGURE 3:**
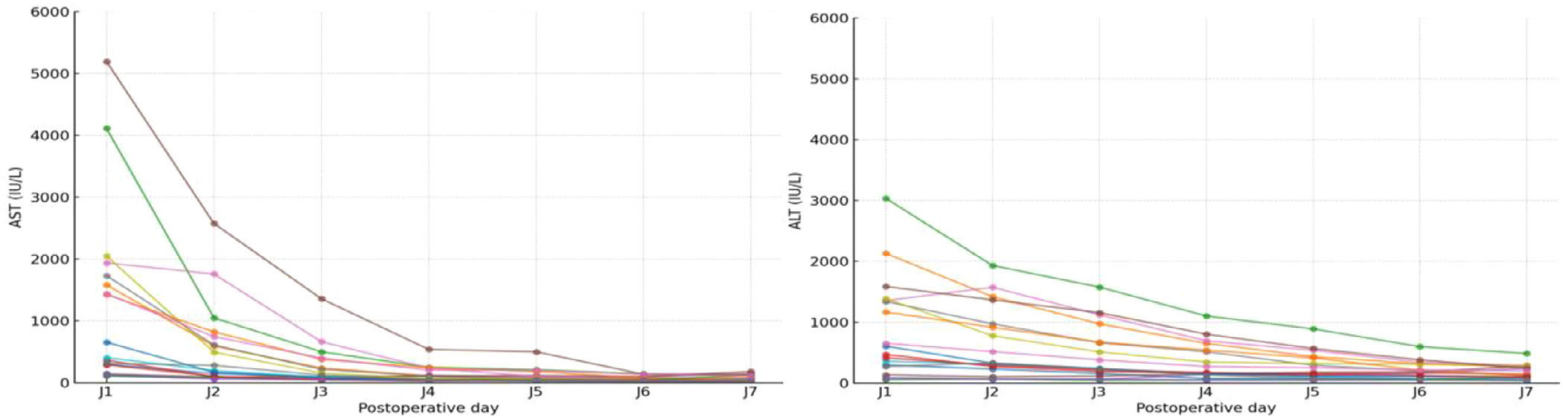
Postoperative evolution of AST and ALT during the first week in recipients of NMP‐preserved grafts. Each line represents an individual patient. ALT, alanine aminotransferase; AST, aspartate aminotransferase.

Overall, the incidences of EAD and PNF were 30.6% and 0%, respectively. EAD occurred in 44.4% of NMP recipients and 26.0% of SCS recipients (*p* = 0.204), with no cases of PNF reported in either group. Peak AST was higher in the NMP group (470 [272–1354] vs. 467 [234–606] IU/L; *p* = 0.047), whereas ALT did not differ (404 [289–2046] vs. 558 [400–874] IU/L; *p* = 0.149). Five grafts met EAD criteria based on AST or ALT >2000 IU/L within the first seven postoperative days. When applying the modified EAD definition, incidence was lower and more comparable between groups (17% for NMP vs. 24% for SCS, *p* = 0.550). The distribution of EAD phenotypes according to preservation method is shown in Figure 2, with a predominance of cytolysis‐driven EAD after NMP. Figure 3 illustrates the dynamics of AST and ALT over the first seven postoperative days in recipients of NMP preserved grafts, showing the early peak followed by a rapid decline. Re‐transplantation occurred in 6.2% overall (5.6% NMP vs. 6.3% SCS; *p* = 0.910). No PNF occurred among NMP grafts meeting viability criteria.

Biliary complications occurred in 38.2% overall (43.8% NMP vs. 36.6% SCS; *p* = 0.684). In NMP recipients, events included 2 non‐anastomotic strictures, 3 anastomotic strictures, and 2 bile leaks all managed by endoscopic or surgical procedures (Table S4).

Notably, grafts associated with EAD or biliary complications were distributed across the full range of perfusion durations, without an apparent clustering toward longer NMP times. Vascular complications were similar (arterial 11.1% vs. 11.1%, *p* = 0.996; venous 5.6% vs. 0%, *p* = 0.087) (Table 2).

Severe postoperative morbidity (CD grade ≥ III, excluding death) was lower in NMP (0% vs. 29.1%; *p* = 0.027). Given the limited sample size, these findings should be interpreted with caution.

Within 90 days, one death occurred in each group (NMP 5.6%, SCS 1.2%; *p* = 0.087). ICU and total hospital stay were similar (ICU 4 [2, 3, 4, 5, 6] versus 4 [2, 3, 4, 5] days, *p* = 0.605; hospital 16 [12, 13, 14, 15, 16, 17, 18, 19, 20, 21, 22, 23, 24, 25, 26, 27, 28, 29, 30, 31, 32, 33] versus 14 [13, 14, 15, 16, 17, 18, 19, 20, 21, 22, 23, 24, 25, 26, 27, 28] days, *p* = 0.838).

Two deaths occurred in the NMP group: one from acute pancreatitis on day 20, and one after early hepatic artery thrombosis with retransplantation and later multiorgan failure.

#### 3.2.2 Death‐Censored Graft Survival

**FIGURE 4:**
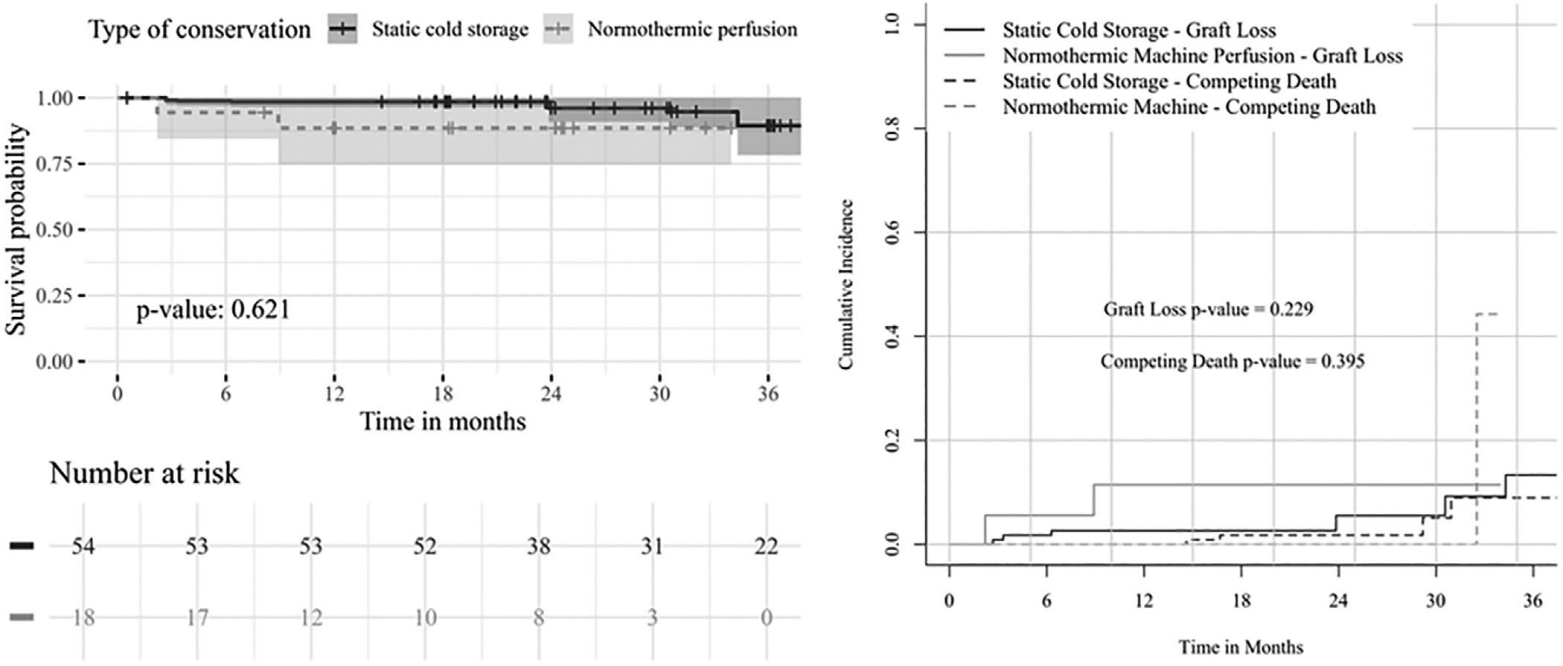
Kaplan–Meier estimates of overall survival (a) and Kalbfleisch—Prentice cumulative incidence of graft loss and competing patient death (b) according to liver conservation for patients undergoing orthotopic liver transplantation.

After a median follow‐up of 23.8 months (IQR 21.2–27.5), death‐censored graft survival at 12 and 24 months was 88.5% [95% CI 0.74–1.00] versus 98.5% [0.96–1.00] (*p* = 0.427) and 88.5% [0.74–1.00] versus 96.0% [0.90–1.00] (*p* = 0.584). The cumulative incidence of graft loss at 24 months was 10.5% for NMP versus 5.6% for SCS (Gray test *p* = 0.229). Death with functioning graft was 0.6% versus 0.1% (*p* = 0.395) (Figure 4). Kaplan–Meier analysis showed no significant difference (log‐rank *p* = 0.621). In univariable Cox analysis, NMP was not significantly associated with graft loss (HR 3.62, 95% CI 0.68–19.3, *p* = 0.13). After adjustment for MELD, HCC indication, and pretransplant treatment, NMP remained not independently associated with graft survival (Cox adjusted HR 2.46, 95% CI 0.38–15.9, *p* = 0.30; Fine‐Gray adjusted sub‐HR 2.30, 95% CI 0.45–14.1, *p* = 0.46). The recipient MELD score was the only independent predictor of graft loss (adjusted HR 0.91, 95% CI 0.84–0.98, *p* = 0.017) (Table 3).

**TABLE 3:** Multivariable Cox‐proportional hazards of overall survival for patients undergoing orthotopic liver transplantation.

		Univariable analysis^a^		Multivariable analysis^a^	
Characteristics	Missing values^b^	HR (95% CI)	*p*‐value	Adjusted HR (95% CI)	*p*‐value
Liver conservation, NMP (vs. static cold storage)	0 (0)	3.62 (0.68,19.3)	0.13	2.46 (0.38, 15.9)	0.3
Liver cold ischemia time, minutes	0 (0)	1.00 (0.99, 1.00)	0.19	1.00 (0.99, 1.00)	0.3
Age, years	0 (0)	1.02 (0.89, 1.17)	0.78		
Sex	0 (0)		0.26		
Indication for LT			0.28		
Cirrhosis		—			
HCC		4.18 (0.32, 54.4)			
Bridge treatment to LT	0 (0)		0.21	—	—
No treatment		—	—	—	—
Treatment		4.11 (0.46, 36.7)		2.57 (0.26, 25.6)	0.4
MELD score	0 (0)	0.89 (0.78, 1.01)	0.073	0.91(0.84, 0.98)	**0.017**
Liver macrovesicular steatosis	0 (0)	0.98 (0.93, 1.04)	0.51	—	—
Sodium (serum) >155 mmol/L	0 (0)		0.27	—	—
No					
Yes		0.26 (0.02, 2.78)			
Occurrence of cardiac arrest before liver procurement	0 (0)		0.61		
No		—	—		
Yes		1.81(0.19, 17.1)			
EAD	0 (0)		0.48		
No		—			
Yes		1.82 (0.35, 9.57)			

Abbreviations: CI: confidence interval; EAD, early allograft dysfunction; HCC, hepatocellular carcinoma; HR, Hazards Ratio; LT, liver transplantation; MELD, model for end‐stage liver disease; NMP, normothermic machine perfusion.

^a^ Risks were computed using multivariable Cox proportional hazards models performed after multiple imputation of liver steatosis missing data. Both univariable and multivariable Cox analyses were performed in each imputed data set (*n* = 20) and the estimates and standard errors were pooled into a final point estimate with robust standard errors according to Rubin's rule.

^b^ Missing values in parentheses are percentages.

## 4 Discussion

This study demonstrated that predefined viability criteria applied during prolonged end‐ischemic NMP reliably and safely identified transplantable liver grafts, achieving outcomes equivalent to SCS and supporting the feasibility and safety of routine clinical use. Among 20 perfused grafts, 18 met the predefined thresholds and were transplanted, while the two that failed were discarded and later confirmed histologically as metabolically non‐viable. The absence of PNF among transplanted grafts confirms the algorithm's safety and its capacity to prevent catastrophic graft failure.

Unlike previous reports focusing on discarded or high‐risk grafts [16, 17, 18, 19, 20], this cohort included only standard DBD livers that would have been transplanted under routine conditions. NMP was primarily implemented for logistical optimization rather than graft rescue. This study therefore addresses a logistics‐driven application of prolonged end‐ischemic NMP in standard DBD LT and should not be extrapolated to DCD rescue settings. The demonstration that NMP achieved comparable EAD, mEAD, and graft survival rates to SCS confirms that normothermic preservation can reproduce standard outcomes while adding the advantage of real‐time viability assessment. These criteria therefore act mainly as a safeguard, excluding non‐viable grafts while maintaining excellent safety.

Although CIT was included as a matching variable, it remained shorter in the NMP group after matching. This residual imbalance may reflect incomplete control of confounding and should therefore be acknowledged when interpreting the comparative outcomes between the NMP and SCS groups.

Lactate clearance emerged as a key safety and exclusion parameter, with a strong negative predictive value for PNF, when interpreted within a composite viability assessment including metabolic and hemodynamic criteria. However, adequate lactate clearance alone does not fully capture graft resilience, as early dysfunction or biliary complications have been reported despite satisfactory metabolic recovery, highlighting the need for complementary markers, such as emerging mitochondrial indicators including flavin mononucleotide, which may further refine viability assessment during machine perfusion. [7, 8, 9, 16, 30, 31, 32]. All grafts fulfilling predefined criteria demonstrated satisfactory posttransplant function, supporting the clinical robustness of these thresholds during prolonged end‐ischemic NMP.

Beyond viability, the early cytolysis pattern observed after NMP, characterized by an AST/ALT peak followed by a rapid decline, is consistent with transient phenomena rather than sustained graft dysfunction. This elevation likely results from enzyme washout during oxygenated reperfusion and localized mechanical compression of the hepatic dome against the perfusion tray. Both have been described in experimental and clinical series and had no clinical consequence in this cohort, as all grafts showed uniform functional recovery [7, 9, 33].These findings refine the interpretation of early biochemical changes observed after NMP. Standard EAD defined by Olthoff criteria was intentionally retained as the primary endpoint to preserve comparability with conventional transplant series, while mEAD was used only as a sensitivity analysis to aid interpretation of early transaminase elevations observed after NMP.

From a technical standpoint, avoidance of prolonged focal compression during NMP may require gentle repositioning of the graft within the perfusion chamber during ongoing perfusion, to prevent sustained pressure on the hepatic dome. In parallel, alternative perfusion platforms developed by the Zurich group [34] incorporate dynamic organ support with periodic motion mimicking diaphragmatic excursions, representing a promising strategy to minimize static mechanical stress during normothermic preservation.

While such metabolic thresholds ensure hepatocellular recovery and prevent non‐function, their clinical benefit extends beyond metabolic safety. In this cohort, severe postoperative morbidity (CD ≥ III) was significantly lower after NMP compared with SCS (0% vs. 29%, *p* = 0.027). However, this finding should be interpreted cautiously given the limited sample size and the small number of events. Although this observation may suggest that functional assessment during NMP facilitates graft selection, the study was not powered to establish a causal relationship between NMP and reduced postoperative morbidity. These findings nonetheless support the potential clinical value of viability assessment during NMP for donor–recipient safety, even when applied to standard DBD transplantation.

However, the same algorithm, entirely hepatocellular‐based, does not evaluate cholangiocellular integrity [24], which may contribute to the observed biliary outcomes. In this study, biliary complications were defined as anastomotic or non‐anastomotic strictures and bile leaks identified during postoperative follow‐up and managed according to standard clinical practice. Their occurrence, despite satisfactory hepatocellular recovery during NMP, likely reflects the absence of dedicated cholangiocellular assessment within the predefined viability criteria rather than graft non‐viability. Hepatocellular recovery during perfusion appears sufficient to predict global graft tolerance and postoperative stability, but not to capture the specific vulnerability of the biliary epithelium. Integrating bile‐derived indicators (pH, bicarbonate, glucose) and molecular markers of cholangiocyte injury into future viability frameworks could provide a more comprehensive view of graft fitness. Such integrative assessment would preserve the safety achieved through current metabolic criteria while expanding their predictive capacity to include biliary outcomes [14, 20].

Future viability frameworks may therefore benefit from incorporating cholangiocellular‐specific parameters, including bile pH, bicarbonate, and glucose, as well as emerging molecular biomarkers of biliary epithelial injury, to complement hepatocellular assessment and improve prediction of biliary outcomes. Minor perfusion‐related events, such as transient hepatic dome compression on the perfusion tray, could contribute to enzyme release without impacting outcomes, emphasizing the importance of procedural refinement rather than biological concern.

By supporting the feasibility of applying predefined thresholds in a real‐world setting, this study reinforces the clinical framework for functional graft assessment. All transplanted grafts meeting the criteria functioned immediately, providing a 100% negative predictive value for PNF. Nonetheless, the absence of correlation with EAD incidence indicates that current criteria remain insufficient to predict the full spectrum of graft performance. Future refinements should focus on dynamic metabolic patterns and the integration of biliary or molecular signatures that better reflect overall graft resilience.

Several limitations must be acknowledged. This was a single‐center study with a small sample size, limiting statistical power to detect moderate differences. The inverse association observed between MELD score and graft loss in the multivariable analysis should be interpreted cautiously. Given the limited number of events, the stability of the model may be limited, and this finding likely reflects statistical instability rather than a true protective effect. Only grafts meeting inclusion criteria were perfused, introducing potential selection bias toward clinically acceptable organs. The exclusive use of DBD grafts restricts generalizability, as the relevance of metabolic thresholds may differ in DCD settings. Despite these constraints, the uniformity of results across preservation strategies and the absence of adverse outcomes confirm that prolonged end‐ischemic NMP can be safely and seamlessly integrated into standard clinical workflows.

In summary, prolonged end‐ischemic NMP using predefined functional criteria provides a safe, reproducible, and physiologically grounded approach for graft assessment before transplantation. While the algorithm effectively prevents PNF, its ability to achieve outcomes equivalent to conventional preservation demonstrates its readiness for routine application in standard DBD transplantation. The comparable biliary outcomes underline the need to complement hepatocellular‐based assessment with cholangiocellular and molecular biomarkers, paving the way toward a more integrative and predictive model of graft viability.

## Funding

No funding was received for this research or its publication.

## Disclosure

This study has not been previously presented or communicated at any meeting or conference.

## Conflicts of Interest

The authors declare no conflicts of interest.

## Supporting information




**Supporting File 1:** ctr70539‐sup‐0001‐SuppMat.docx

## Data Availability

Data supporting the findings of this study are available from the corresponding author upon reasonable request and after approval by the institutional ethics committee.

## References

[1] C. D. L. Ceresa , D. Nasralla , C. C. Coussios , et al., “The Case for Normothermic Machine Perfusion in Liver Transplantation,” Liver Transplantation 24, no. 2 (2018): 269–275. 29272051 10.1002/lt.25000

[2] P. N. Martins , J. E. Buchwald , H. Mergental , et al., “The Role of Normothermic Machine Perfusion in Liver Transplantation,” International Journal of Surgery 82S (2020): 52–60. 32417462 10.1016/j.ijsu.2020.05.026

[3] M. C. Nguyen , C. Zhang , Y. H. Chang , et al., “Improved Outcomes and Resource Use With Normothermic Machine Perfusion in Liver Transplantation,” JAMA Surgery 160, no. 3 (2025): 322–330. 39878966 10.1001/jamasurg.2024.6520 PMC11780509

[4] A. Schlegel , H. Mergental , C. Fondevila , et al., “Machine Perfusion of the Liver and Bioengineering,” Journal of Hepatology 78, no. 6 (2023): 1181–1198. 37208105 10.1016/j.jhep.2023.02.009

[5] B. Lascaris , V. E. de Meijer , and R. J. Porte , “Normothermic Liver Machine Perfusion as a Dynamic Platform for Regenerative Purposes: What Does the Future Have in Store for Us?,” Journal of Hepatology 77, no. 3 (2022): 825–836. 35533801 10.1016/j.jhep.2022.04.033

[6] H. Mergental , R. W. Laing , J. Hodson , et al., “Introduction of the Concept of Diagnostic Sensitivity and Specificity of Normothermic Perfusion Protocols to Assess High‐Risk Donor Livers,” Liver Transplantation 28, no. 5 (2022): 794–806. 34619014 10.1002/lt.26326

[7] C. J. E. Watson , R. Gaurav , C. Fear , et al., “Predicting Early Allograft Function After Normothermic Machine Perfusion,” Transplantation 106, no. 12 (2022): 2391–2398. 36044364 10.1097/TP.0000000000004263 PMC9698137

[8] H. Jeddou , S. Tzedakis , M. A. Chaouch , et al., “Viability Assessment During Normothermic Machine Liver Perfusion: A Literature Review,” Liver International 45, no. 2 (2025): e16244. 39821671 10.1111/liv.16244 PMC11740183

[9] C. J. E. Watson , V. Kosmoliaptsis , C. Pley , et al., “Observations on the ex situ Perfusion of Livers for Transplantation,” American Journal of Transplantation 18, no. 8 (2018): 2005–2020. 29419931 10.1111/ajt.14687 PMC6099221

[10] M. E. Sutton , S. op den Dries , N. Karimian , et al., “Criteria for Viability Assessment of Discarded Human Donor Livers During ex vivo Normothermic Machine Perfusion,” PLoS One 9, no. 11 (2014): e110642. 25369327 10.1371/journal.pone.0110642 PMC4219693

[11] H. Mergental , B. T. F. Stephenson , R. W. Laing , et al., “Development of Clinical Criteria for Functional Assessment to Predict Primary Nonfunction of High‐Risk Livers Using Normothermic Machine Perfusion,” Liver Transplantation 24, no. 10 (2018): 1453–1469. 30359490 10.1002/lt.25291 PMC6659387

[12] A. Parente , F. Tirotta , A. Pini , et al., “Machine Perfusion Techniques for Liver Transplantation—A Meta‐Analysis of the First Seven Randomized‐Controlled Trials,” Journal of Hepatology 79, no. 5 (2023): 1201–1213. 37302578 10.1016/j.jhep.2023.05.027

[13] A. P. M. Matton , Y. de Vries , L. C. Burlage , et al., “Biliary Bicarbonate, pH, and Glucose Are Suitable Biomarkers of Biliary Viability During ex situ Normothermic Machine Perfusion of Human Donor Livers,” Transplantation 103, no. 7 (2019): 1405–1413. 30395120 10.1097/TP.0000000000002500 PMC6613725

[14] I. E. M. de Jong , S. B. Bodewes , O. B. van Leeuwen , et al., “Restoration of Bile Duct Injury of Donor Livers During Ex Situ Normothermic Machine Perfusion,” Transplantation 107, no. 6 (2023): e161–e172. 36721302 10.1097/TP.0000000000004531 PMC10205124

[15] A. M. Thorne , J. C. Wolters , B. Lascaris , et al., “Bile Proteome Reveals Biliary Regeneration During Normothermic Preservation of Human Donor Livers,” Nature Communications 14, no. 1 (2023): 7880. 10.1038/s41467-023-43368-y PMC10689461 38036513

[16] H. Mergental , R. W. Laing , A. J. Kirkham , et al., “Transplantation of Discarded Livers Following Viability Testing With Normothermic Machine Perfusion,” Nature Communications 11, no. 1 (2020): 2939. 10.1038/s41467-020-16251-3 PMC7298000 32546694

[17] C. Quintini , L. Del Prete , and A. Simioni , “Transplantation of Declined Livers After Normothermic Perfusion,” Surgery 171, no. 3 (2022): 747–756. 35065791 10.1016/j.surg.2021.10.056

[18] F. C. Olumba , F. Zhou , Y. Park , et al., “Normothermic Machine Perfusion for Declined Livers: A Strategy to Rescue Marginal Livers for Transplantation,” Journal of the American College of Surgeons 236, no. 4 (2023): 614–625. 36728302 10.1097/XCS.0000000000000555

[19] O. B. van Leeuwen , S. B. Bodewes , and V. A. Lantinga , “Sequential Hypothermic and Normothermic Machine Perfusion Enables Safe Transplantation of High‐Risk Donor Livers,” American Journal of Transplantation 22, no. 6 (2022): 1658–1670. 35286759 10.1111/ajt.17022 PMC9325426

[20] Q. Liu , L. Del Prete , and K. Ali , “Sequential Hypothermic and Normothermic Perfusion Preservation and Transplantation of Expanded Criteria Donor Livers,” Surgery 173, no. 3 (2023): 846–854. 36302699 10.1016/j.surg.2022.07.035

[21] P. Viana , S. Castillo‐Flores , M. M. R. Mora , et al., “Normothermic Machine Perfusion vs. Static Cold Storage in Liver Transplantation: A Systematic Review and Meta‐Analysis,” Artificial Organs 49, no. 6 (2025): 945–954. 39887468 10.1111/aor.14960

[22] M. Kalisvaart , J. E. de Haan , W. G. Polak , et al., “Comparison of Postoperative Outcomes Between Donation After Circulatory Death and Donation After Brain Death Liver Transplantation Using the Comprehensive Complication Index,” Annals of Surgery 266, no. 5 (2017): 772–778. 28742700 10.1097/SLA.0000000000002419

[23] H. Mergental , R. W. Laing , A. J. Kirkham , et al., “Discarded Livers Tested by Normothermic Machine Perfusion in the VITTAL Trial: Secondary End Points and 5‐Year Outcomes,” Liver Transplantation 30, no. 1 (2024): 30–45. 38109282 10.1097/LVT.0000000000000270

[24] K. M. Olthoff , L. Kulik , B. Samstein , et al., “Validation of a Current Definition of Early Allograft Dysfunction in Liver Transplant Recipients and Analysis of Risk Factors,” Liver Transplantation 16 (2010): 943–949. 20677285 10.1002/lt.22091

[25] J. L. Martin , F. Rhodes , S. Upponi , et al., “Localized Liver Injury During Normothermic ex situ Liver Perfusion Has No Impact on Short‐Term Liver Transplant Outcomes,” Transplantation 108, no. 6 (2024): 1403–1409. 38419153 10.1097/TP.0000000000004970 PMC11115454

[26] J. A. Richards , R. Gaurav , S. S. Upponi , et al., “Machine Perfusion‐Leaving Its Mark on Liver Transplantation,” Transplantation 105, no. 2 (2021): e28–e29. 33492117 10.1097/TP.0000000000003462

[27] H. Hartog , A. Hann , and M. Perera , “Primary Nonfunction of the Liver Allograft,” Transplantation 106, no. 1 (2022): 117–128. 33982912 10.1097/TP.0000000000003682

[28] D. Dindo , N. Demartines , and P. A. Clavien , “Classification of Surgical Complications: A New Proposal With Evaluation in a Cohort of 6336 Patients and Results of a Survey,” Annals of Surgery 240, no. 2 (2004): 205–213. 15273542 10.1097/01.sla.0000133083.54934.ae PMC1360123

[29] S. van Buuren and K. Groothuis‐Oudshoorn , “mice: Multivariate Imputation by Chained Equations in R,” Journal of Statistical Software 45 (2011): 1–67.

[30] J. Hofmann , A. T. Meszaros , A. Butler , et al., “Predictive Value of Early Postoperative Lactate (<6 h) During Normothermic Machine Perfusion and Outcome After Liver Transplantation: Results From a Multicentre Study,” British Journal of Surgery 111, no. 6 (2024): znae084, 10.1093/bjs/znae084, Erratum in: *British Journal of Surgery* 111, no. 7 (2024): znae182. 38875136 PMC11177788

[31] D. Ghinolfi , E. Rreka , V. De Tata , et al., “Pilot, Open, Randomized, Prospective Trial for Normothermic Machine Perfusion Evaluation in Liver Transplantation from Older Donors,” Liver Transplantation: Official Publication of the American Association for the Study of Liver Diseases and the International Liver Transplantation Society 25, no. 3 (2019): 436–449. 30362649 10.1002/lt.25362

[32] R. Panconesi , M. Flores Carvalho , M. Mueller , et al., “Viability Assessment in Liver Transplantation‐What Is the Impact of Dynamic Organ Preservation?,” Biomedicines 9, no. 2 (2021): 161. 33562406 10.3390/biomedicines9020161 PMC7915925

[33] M. Bral , N. Aboelnazar , S. Hatami , et al., “Clearance of Transaminases During Normothermic ex situ Liver Perfusion,” PLoS One 14, no. 4 (2019): e0215619. 31017974 10.1371/journal.pone.0215619 PMC6481840

[34] D. Eshmuminov , D. Becker , L. Bautista Borrego , et al., “An Integrated Perfusion Machine Preserves Injured Human Livers for 1 Week,” Nature Biotechnology 38, no. 2 (2020): 189–198. 10.1038/s41587-019-0374-x PMC7008032 31932726

