# Supplementary material for: Performance of Predefined Viability Criteria During Prolonged End‐Ischemic Normothermic Perfusion of Brain‐Dead Donor Liver Grafts
Source: Clin Transplant. 2026 Apr 25;40:e70539. doi: 10.1111/ctr.70539 (PMC13109906; doi:10.1111/ctr.70539)
Supplement: Supplementary file 1 — Supporting File 1: ctr70539‐sup‐0001‐SuppMat.docx [file CTR-40-e70539-s001.docx]

**Supplementary Material**

**Performance of predefined viability criteria during prolonged normothermic liver perfusion**

Heithem Jeddou^1,2^, Stylianos Tzedakis^3^, Michel Samson^2^, Karim Boudjema^1,2^

**Table of contents**

Supplementary Methods.............................................................................................................2

Supplementary figures................................................................................................................5

Supplementary tables..................................................................................................................8

**Supplementary Methods**

**Study Protocol**

The trial was conducted at Rennes University Hospital. Donor hepatectomy time was recorded by the independent organ procurement coordinator and communicated to the on-call liver transplant surgeon. Livers were assigned to undergo normothermic machine perfusion (NMP) if donor hepatectomy was completed between 16:00 and 00:00. Grafts were preserved using static cold storage (SCS) and transplanted immediately if the hepatectomy was completed between 00:01 and 15:59. The protocol did not interfere with the standard organ allocation and acceptance procedures.

**Procurement and Machine Perfusion Settings**

All liver grafts were retrieved using standard multiorgan procurement procedures and flushed in situ with cold CUSTODIOL® solution. During transportation to the transplant center, grafts were preserved in static cold storage using CUSTODIOL®. Since cold ischemia time exceeded 3 hours in all cases, the procedure was classified as post-static cold storage (post-SCS) NMP.

Upon arrival, a standard back-table procedure was performed, including dissection and cannulation of the portal vein, hepatic artery, inferior vena cava, and bile duct. The OrganOx Metra® normothermic perfusion system was used for all perfusions. A sterile disposable circuit was installed and primed with 500 mL of Gelofusine and 3 units of donor-matched packed red blood cells. The perfusate was supplemented with insulin, heparin, prostacyclin, calcium gluconate, magnesium sulfate, sodium bicarbonate (added incrementally to maintain pH between 7.35 and 7.45), antibiotics, bile salts, and fat-free parenteral nutrition (Nutriflex®). Before connection to the device, liver grafts were flushed with 1 L of Gelofusine to remove residual preservation solution. Perfusion was initiated in the operating room and continued until recipient hepatectomy was complete. Prior to implantation, all livers were flushed with cold HTK solution.

The OrganOx Metra® device maintained a target temperature of 37°C and continuously regulated perfusion pressure, blood gases, and flow. Typical flow rates were 200–400 mL/min through the hepatic artery and 1,000–1,200 mL/min through the portal vein. Inferior vena cava pressure was maintained between 0 and 2 mmHg. Glucose was manually measured every 4 hours and entered into the system; if levels dropped below 10 mmol/L, Nutriflex® infusion was automatically adjusted.

Perfusion duration ranged from 4 to 24 hours, depending on surgical scheduling. Although the primary aim of the study was logistical rather than functional assessment, all grafts were expected to meet minimal viability criteria before implantation.

**Monitoring and Viability Parameters**

Perfusate samples were analyzed hourly for lactate concentration. Perfusate pH was continuously monitored. Glucose was manually measured every 4 hours. Bile output was collected and quantified hourly. Hemodynamic stability (arterial and portal flow rates) was monitored in real-time throughout perfusion.

A graft was considered viable if lactate decreased to ≤2.5 mmol/L within 4 hours of NMP initiation, and if at least two of the following additional criteria were met: sustained bile production, perfusate pH ≥7.30, glucose metabolism, stable arterial and portal flows (≥150 and ≥500 mL/min, respectively), and homogeneous macroscopic appearance with soft parenchymal consistency.

**Liver Transplantation and Postoperative Care**

All patients underwent orthotopic liver transplantation with preservation of the inferior vena cava. After standard laparotomy, the liver hilum was dissected and the native liver was removed. The graft was flushed with 500 mL of 5% albumin before implantation. Vascular reconstruction included side-to-side caval anastomosis, end-to-end portal vein anastomosis, followed by hepatic artery and bile duct reconstruction.

Immunosuppression consisted of tacrolimus, mycophenolate mofetil, and a short course of corticosteroids. Doppler ultrasonography was systematically performed on postoperative days 1 and 7, or earlier if dysfunction was suspected. When indicated, contrast-enhanced CT imaging was performed and therapeutic decisions were discussed in multidisciplinary meetings. Patients were discharged once liver function was stable, immunosuppression was within therapeutic range, and sufficient autonomy was achieved.

**Control Group**

Livers preserved in static cold storage (SCS) were retrieved, transported, and transplanted according to institutional standard practice without modification.

**Supplementary figures**


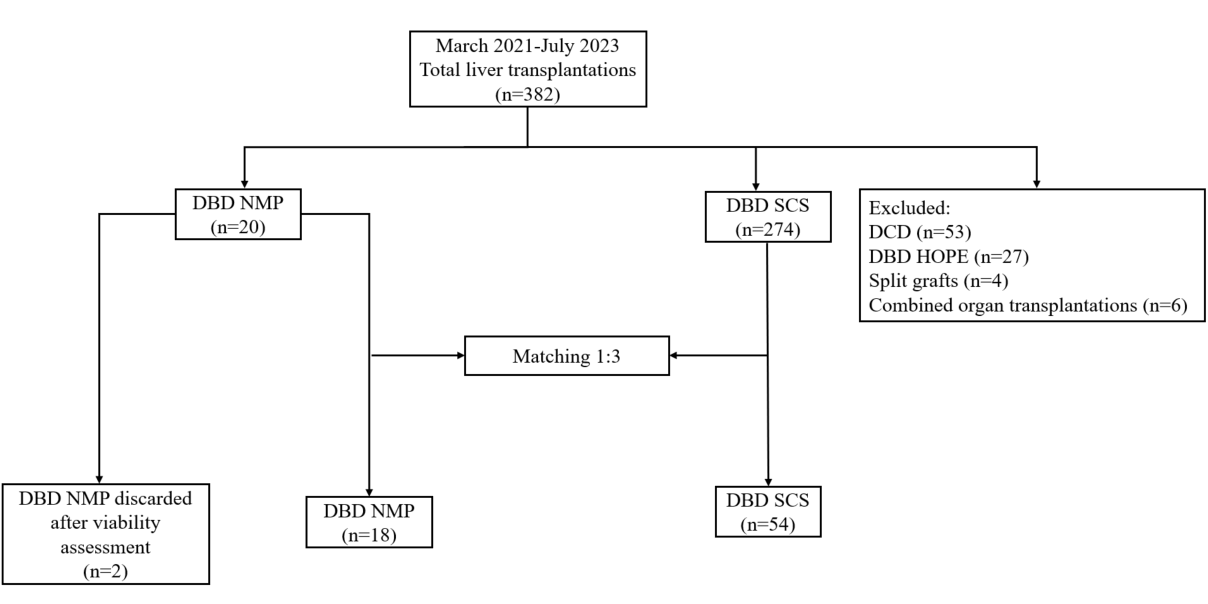


**Figure S1: Patient flowchart**


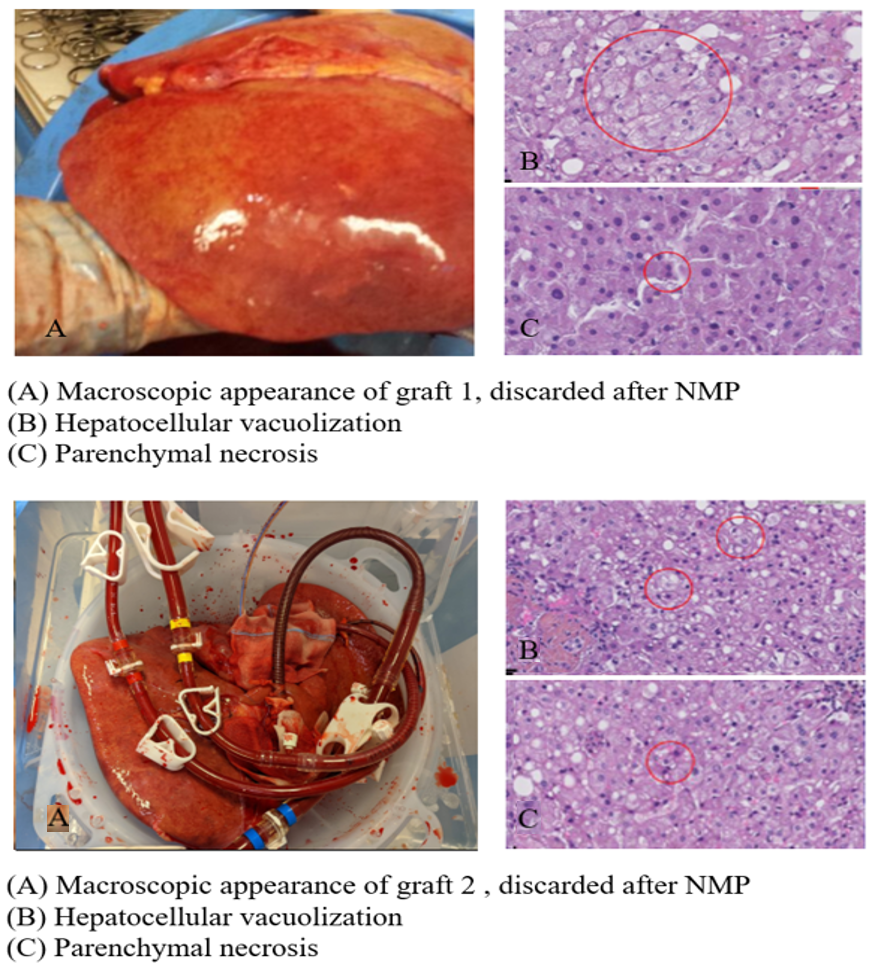


**Figure S2: Macroscopic and histological findings of two grafts discarded after NMP**

**
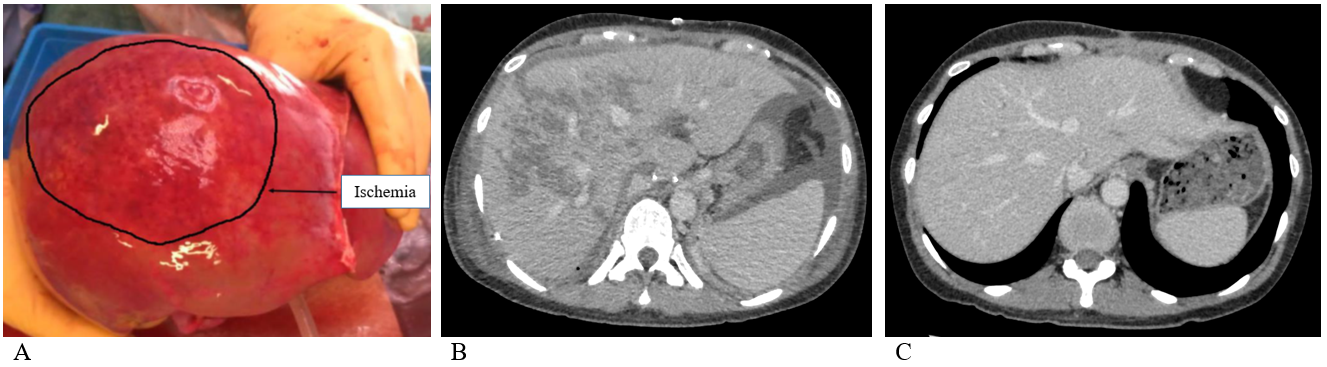
**

**Figure S3: (A)** Intraoperative view showing an ischemic area (outlined) on the superior-posterior segments, caused by the weight of the graft resting on the perfusion tray during NMP. **(B)** Postoperative CT scan on day 1 showing a corresponding hypodense area. **(C)** Control CT scan at 5 weeks demonstrating complete resolution of the ischemic lesion.

**Supplementary tables**

Tableau S1 Individual preservation time components for transplanted NMP grafts (n = 18)

| **NMP graft** | **Cold ischemia time before NMP (min)** | **NMP duration (min)** | **Post-NMP cold ischemia (min)** | **Total preservation time (min)** |
| --- | --- | --- | --- | --- |
| 1 | 250 | 840 | 0 | 1090 |
| 2 | 300 | 720 | 0 | 1020 |
| 3 | 260 | 680 | 0 | 940 |
| 4 | 280 | 760 | 0 | 1040 |
| 5 | 240 | 800 | 0 | 1040 |
| 6 | 310 | 650 | 0 | 960 |
| 7 | 270 | 720 | 0 | 990 |
| 8 | 290 | 690 | 0 | 980 |
| 9 | 230 | 780 | 0 | 1010 |
| 10 | 260 | 740 | 0 | 1000 |
| 11 | 320 | 610 | 0 | 930 |
| 12 | 300 | 660 | 0 | 960 |
| 13 | 280 | 700 | 0 | 980 |
| 14 | 250 | 820 | 0 | 1070 |
| 15 | 270 | 730 | 0 | 1000 |
| 16 | 290 | 680 | 0 | 970 |
| 17 | 240 | 760 | 0 | 1000 |
| 18 | 260 | 710 | 0 | 970 |

**Table S2: Viability criteria for determining liver viability at 240 mins NMP**

| Perfusate lactate | ≤ 2.5 mmol/L |
| --- | --- |
| Bile production | Present |
| Perfusate pH | ≥ 7.30 |
| Glucose metabolism | Active consumption |
| Arterial flow | ≥ 150 mL/min |
| Portal flow | ≥ 500 mL/min |
| Visual assessment | Homogenous perfusion and soft parenchymal consistency |

**Table S3: Individual viability parameters of livers perfused at 240 mins NMP**

| Liver number | Lactate | pH | Bile production | Glucose metabolism | Arterial flow mL/min | Portal flow mL/min | Homogenous perfusion |
| --- | --- | --- | --- | --- | --- | --- | --- |
| Livers meeting viability criteria and transplanted | | | | | | | |
| 1 | 1.2 | 7.25 | Yes | Yes | 540 | 1150 | Yes |
| 2 | 2.3 | 7.22 | No | Yes | 370 | 1140 | Yes |
| 3 | 2 | 7.26 | No | Yes | 610 | 1180 | No |
| 4 | 1 | 7.31 | Yes | Yes | 510 | 1040 | Yes |
| 5 | 1.4 | 7.29 | Yes | Yes | 310 | 1170 | Yes |
| 6 | 1 | 7.33 | Yes | Yes | 740 | 1010 | Yes |
| 7 | 1.8 | 7.37 | No | Yes | 670 | 880 | Yes |
| 8 | 1.6 | 7.36 | Yes | Yes | 620 | 880 | Yes |
| 9 | 1.49 | 7.27 | Yes | Yes | 870 | 1060 | Yes |
| 10 | 0.84 | 7.32 | Yes | Yes | 570 | 1100 | Yes |
| 11 | 1.3 | 7.46 | Yes | Yes | 520 | 1060 | Yes |
| 12 | 0.9 | 7.35 | No | Yes | 620 | 1130 | Yes |
| 13 | 0.8 | 7.32 | Yes | Yes | 610 | 1100 | Yes |
| 14 | 1.1 | 7.29 | No | Yes | 510 | 990 | Yes |
| 15 | 1.4 | 7.36 | Yes | Yes | 480 | 1050 | Yes |
| 16 | 0.6 | 7.34 | Yes | Yes | 550 | 1200 | Yes |
| 17 | 1.4 | 7.35 | Yes | Yes | 540 | 1050 | Yes |
| 18 | 1.1 | 7.34 | Yes | Yes | 550 | 1620 | Yes |
| Livers not meeting viability criteria and not transplanted | | | | | | | |
| 1 | 2.89 | 7.12 | Yes | Yes | 490 | 1030 | No |
| 2 | 2.64 | 7.20 | No | Yes | 650 | 1610 | No |

**Table S4: Types and management of biliary complications in the NMP group**

| **Type of complication** | **Number of patients** | **Management** |
| --- | --- | --- |
| Non-anastomotic strictures | 2 | 1 ERCP  1 Roux-en-Y hepaticojejunostomy |
| Anastomotic strictures | 3 | All managed by ERCP |
| Bile leaks | 2 | 1 Surgical drainage  1 Roux-en-Y hepaticojejunostomy |

ERCP : Endoscopic Retrograde Cholangiopancreatography
